# A Study on *MTHFR* C677T Gene Polymorphism and Alcohol Dependence among *Meiteis* of Manipur, India

**DOI:** 10.1155/2014/310241

**Published:** 2014-10-01

**Authors:** Huidrom Suraj Singh, Kabita Salam, Kallur Nava Saraswathy

**Affiliations:** ^1^Molecular Anthropology Laboratory, Department of Anthropology, University of Delhi, Delhi 110007, India; ^2^Department of Anthropology and Tribal Development, Guru Ghasidas Vishwavidyalaya, Bilaspur, Chhattisgarh 495009, India; ^3^The INCLEN Trust International, Okhla Industrial Area, Phase I, New Delhi 110020, India

## Abstract

Chronic alcohol consumption is reported to be associated with increase in plasma homocysteine levels which is further influenced by the polymorphism in methylenetetrahydrofolate reductase (*MTHFR*) gene. The present study aims to understand the extent of the *MTHFR* C677T polymorphism in alcohol dependent (AD) cases of *Meiteis* of Manipur, a Mendelian population of India. *MTHFR* C677T polymorphism was screened in 313 controls and 139 alcohol dependent (AD) cases who all met DSM-IV criteria for alcohol dependence. Both AD cases and controls were unrelated up to 1st cousin. Among the control group, different drinking patterns like abstainer/nondrinkers (NDs), occasional drinkers (ODs), and moderate drinkers (MDs) are included. Both the groups were found to be in Hardy-Weinberg equilibrium (*P* > 0.05). Genotypic and allelic frequency distribution of *MTHFR* C677T polymorphism did not differ significantly between AD cases and controls (*P* > 0.05). However, individuals carrying mutant (T) allele show more than 1-fold increased risk for AD though not significant (OR = 1.43; 95% CI 0.41–5.01, *P* > 0.05). In conclusion, *MTHFR* C677T polymorphism is not found to be risk marker for AD in present studied population. However, higher prevalence of the mutant T allele may exacerbate deleterious health risk in future especially among alcohol drinkers.

## 1. Introduction

Alcoholism or alcohol dependence (AD) is a complex trait characterized by a cluster of physiological, behavioural, and cognitive phenomena in which the use of alcohol takes on a much higher priority for a given individual than other behaviours that once had greater value [1]. The World Health Organization (WHO) estimates that about 140 million people throughout the world suffer from AD [2]. Moreover, it is one of the leading health risks and is likely to become world's third largest risk factor for disease and disability. Approximately 2.5 million deaths each year are attributable to alcohol, resulting in 4% of all deaths worldwide [3].

Several studies show significance of genetic influences in substance abuse and dependence [4, 5]. However, comprehensive molecular and pathophysiological mechanism associated with AD is not completely revealed yet. As genetic predisposition to AD and its related morbidity and mortality is undebatable, the emerging research has picked up momentum targeting various pathways involved in alcohol consumption and metabolism for deeper understanding of the pathophysiology of AD. Recent studies revealed association of gene that encodes the methylenetetrahydrofolate reductase (*MTHFR* C677T) with AD. However, findings showed inconsistent results [6–8]. Available literature indicates that this polymorphism confers elevation of plasma Hcy levels leading to a condition known as hyperhomocysteinemia [9, 10]. Moreover, chronic alcohol consumption is also often found to be associated with hyperhomocysteinemia [11]. Elevation of Hcy levels may further lead to or be associated with several complex diseases or clinical conditions including cardiovascular diseases, pregnancy complications, neural tube defects, Alzheimer disease, end-stage renal disease, schizophrenia, and noninsulin dependent diabetes, as evidenced from several studies [12, 13].

We therefore believe that studying* MTHFR* C677T gene polymorphism might possibly be helpful for understanding pathophysiology of AD and its associated complex clinical conditions particularly among those populations where prevalence of alcohol consumption/drinking is very high. But no such trial studies have so far been carried out in any Indian community (according to our knowledge). Thus, in the present study, an attempt is made to understand the extent of* MTHFR* C677T gene polymorphism in AD cases and controls from* Meiteis *of Manipur, where prevalence/occurrence of alcohol drinkers is very high [2, 14].

## 2. Methods

### 2.1. General Characteristics

The present study's* Meitei* population consisted of 452 subjects, of which 139 were AD cases and 313 were unrelated, healthy controls matched for ethnicity and geography. All the subjects included in the present study were recruited from four districts of Manipur, namely, Imphal East, Imphal West, Thoubal, and Bishnupur, where* Meiteis* are the predominant inhabitants. Diagnosis of alcohol dependence was made on the basis of Diagnostic and Statistical Manual IV (DSM-IV) criteria for AD [15]. The selected control group included individuals consuming alcohol (occasional and moderate drinkers) who were not categorized as alcohol dependent according to the DSM-IV criteria; 121 were absolute nondrinkers. The present study was approved by the departmental ethics committee, Department of Anthropology, University of Delhi, India. Informed written consent was obtained from all the subjects before collecting the samples.

### 2.2. Genetic Analysis

5 mL of intravenous blood samples were collected in ethylenediaminetetraacetic acid coated vacutainers from all the subjects recruited in the present study using disposable single-use syringes. DNA was isolated from the collected samples using standard protocol [17]. Identification of* MTHFR* C677T polymorphism was performed using previously described protocol using* Hinf*I restriction enzyme digestion [16]. The digested PCR products were observed on a 3% agarose gel electrophoresis stained with ethidium bromide. Positive and negative controls are run along with samples as quality control. Genotyping details of the selected SNP are shown in Table 1.

### 2.3. Statistical Analysis

Genotypic and allele frequencies were calculated by gene counting method and Hardy-Weinberg equilibrium (HWE) was determined using the *χ* ^2^ goodness-of-fit test using POPGENE 1.31 [18]. Genotypic and allelic frequency distribution was assessed among the studied population and comparison was made between NDs and ODs, NDs and MDs, and NDs and AD cases using *χ* ^2^ test. Association of drinking status as well as* MTHFR* C677T allelic and genotypic counts with AD was evaluated by Pearson's *χ* ^2^-test followed by odds ratio (OR) at 95% confidential interval (CI), using the freely available 2 × 2 contingency table [19]. Statistical significance was considered at 5% level. Power of the study was calculated using online available software (http://osse.bii.a-star.edu.sg/index.php).

## 3. Results

In the present study, a comparative analysis of the selected SNP,* MTHFR* C677T polymorphism was carried out among alcohol dependent (AD) cases and controls to understand the effects and their outcome. However, the selected control group included nondrinkers (NDs) and individuals consuming alcohol who were not categorized as alcohol dependence as per DSM-IV criteria, rather they were occasional drinkers (ODs) and moderate drinkers (MDs). To control the biasness, selected genetic marker was assessed in the subgroups of controls, that is, NDs, ODs, MDs, and AD cases (see Table 2).

Less than 3% of individuals were found to have* MTHFR* 677TT mutant homozygous genotype among the studied* Meitei* population. The genotypic frequency distribution of the* MTHFR* 667CC, 667CT, and 667TT genotypes were 65.47%, 31.65%, and 2.88%, respectively, among AD cases compared with 72.84%, 24.92%, and 2.24%, respectively, among controls. Both groups, that is, AD cases and controls, were found to be in Hardy-Weinberg equilibrium (*P* > 0.05). No significant difference is observed between AD cases and combined controls with respect to genotypic and allelic frequency distribution of* MTHFR* C677T polymorphism (*P* > 0.05). Relative risk analysis reveals that individuals who carry mutant T allele in both heterozygous or homozygous condition show more than onefold increased risk for AD (OR = 1.43, CI 0.41–5.01 and OR = 1.41, CI 0.91–2.20, resp.); however, the risk was not found to be statistically significant (*P* > 0.05). Power of the study was found to be <20%, which could possibly be due to smaller sample size of the studied population.

To assess the extent of* MTHFR* C677T polymorphism on different patterns of drinking, mutant homozygous 677TT genotype was further assessed in the three subgroups of controls (NDs, ODs, and MDs) and AD cases. 677TT genotype individuals were found to be lower in NDs (2.48%) as compared to ODs (3.54%) and AD cases (2.88%); the difference was not found to be statistically significant (*P* > 0.05). Though 677TT genotype is found to be completely absent among MDs. This could possibly be due to smaller sample size among the moderate drinkers (MDs). Distribution of the mutant T allele frequency showed an increasing trend from NDs to ODs to AD cases with slight drop among MDs.

To understand the relative risk of* MTHFR* 677TT genotype on different patterns of drinking, odds ratio was calculated for ODs, MDs, and AD cases taking NDs as reference group. Except AD cases (OR = 1.47, CI 0.86–2.50), other groups, that is, ODs (OR = 1.10, CI 0.62–1.95) and MDs (OR = 1.01, CI 0.53–1.91) showed more or less than onefold increased risk for alcohol dependence, though risks were not found to be statistically significant (*P* > 0.05) in all the three alcohol drinking categories (Table 3).

## 4. Discussion

Alcohol is a toxic substance that can affect each and every organ of human body particularly stomach, liver, brain, and heart. Alcohol has been found to interact with* MTHFR* gene polymorphism as indicated by several literatures in modifying the risk of several complex diseases including cardiovascular diseases, diabetes, colon cancer, breast cancer, and hepatocellular carcinoma [20, 21]. Moreover, previous studies have associated* MTHFR* gene polymorphism with decreased enzymatic activity and modified homocysteine regulation. Therefore, chronic alcohol consumption may increase the risk of causing/developing total plasma homocysteine levels (i.e., hyperhomocysteinemia) which may further lead to several disease phenotypes in the presence of the reduced* MTHFR* activity associated with the polymorphic gene. The present study determines and compares* MTHFR* C677T polymorphism among AD cases and controls among* Meiteis* of Manipur, which is reported to have higher prevalence of alcohol dependence [2, 14]. One of the contributing factors of alcohol dependence in Manipur has been cited as widely available in every nook and corner despite the fact that it is officially declared “dry” state.

The* MTHFR* C677T polymorphism has been investigated for its association with several complex diseases in several studies across different populations; however, results are not consistent. One of the possible reasons for this could be attributed to variation in allelic frequency distribution in different population groups. The 677T allele frequency is found to be highest in European populations ranging from 24.1% to 64.3% and, hence, it is presumed to be originated in Europe in the late state of human evolution. However, zero frequency of this allele is reported from African population [22]. In Indian population, distribution of 677T allele ranges from complete absence to 23.7% and highest frequency was reported from North-Indian population. Moreover, frequency of T allele is found to be relatively higher among caste populations as compared to that of tribal populations of India. Linguistically, Indo-European speakers have relatively higher T allele frequency followed by Tibeto-Burman, Dravidian, and Austro-Asiatic speakers [22].

An association study carried out in European Caucasoid population demonstrated significant association of mutant T allele of* MTHFR* C677T polymorphism with alcoholic patients with a history of withdrawal symptoms (WS) (T allele frequency − controls = 0.28, AD cases = 0.39; *P* = 0.03) [6]. Similar findings were also reported by Benyamina et al. suggesting possible role of C677T SNP on the etiology of AD. The* MTHFR *TT genotype was found to be more prevalent in AD patients with milder alcohol dependence (Babor type A) and with Lesch type 3, associated with depression [8]. In the present study, no association was found between alleles or genotypes of* MTHFR* C677T polymorphism and AD among* Meiteis* of Manipur. However, our observation of TT genotype being slightly higher among AD cases (2.88) as compared to controls (2.24) is in concurrence with the above association studies where an excess of mutant homozygotes has been reported among the AD cases. Though, no significant difference was observed in the present study. 677T allele frequency distribution among nondrinkers (NDs) that is 0.14 is in accordance with previous report [23]. Moreover, distribution of the mutant T allele frequency showed an increasing trend in the present study from NDs to ODs to AD cases with slight drop among MDs. Lower frequency of T allele among the MDs could be attributed to smaller sample size as well as complete absence of mutant homozygous TT genotype. Such similar increasing trend of T allele is also reported among different subgroups of alcohol use disorders like from healthy controls (0.28) to alcoholic patients with mild withdrawal symptoms (0.33) up to 0.40 in alcohol dependent patients having withdrawal symptoms [6].

However, in contrast to the present study and other association studies reporting association of* MTHFR* C677T gene polymorphism with AD, Saffroy et al. reported a contradictory result among the Caucasian French populations [7]. In fact, they attempted to understand the prevalence of the* MTHFR* C677T polymorphism in AD subjects and its influence on symptoms associated with alcoholism among Caucasian French populations. They observed a significant decrease in* MTHFR* 677TT prevalence among AD cases (9%, 21/242) compared to controls (18%, 17/93) (*P* < 0.02) with an odds ratio of 0.42 for alcoholism in subjects with TT genotype (95% CI 0.21–0.83). They reported that* MTHFR* 677TT genotype could play a protective role against alcohol dependence, for the first time. They also suggested possibility of influence of* MTHFR* C677T polymorphism on the development of the dependence process itself. However, more association studies are needed to really understand whether* MTHFR* C677TT genotype is protective against AD or not. Though no confirmatory studies have been reported till now, one of the possible reasons for such inconsistent results could be attributed to its associations with one-carbon metabolism pathway, as* MHFR* C677T polymorphism plays an important role in elevation of plasma Hcy levels. Moreover, it is also evident that chronic alcohol consumption is associated with folate deficiency which may further lead to an impaired metabolism of homocysteine via inhibiting methionine synthase resulting in the elevation of Hcy level [24]. Both elevation of Hcy level and deficiency of folate may further influence DNA methylation rate, since folate acts as a methyl donor (via S-adenosylmethionine) for DNA methyltransferase and histone methyltransferase [25]. Aberration in DNA methylation status can lead to alterations in reprogramming of epigenetic patterns thereby affecting gene regulation [26]. Further, it also alters the dopaminergic neurotransmission system in the brain affecting cognitive behaviours and addiction [27]. Therefore, polymorphism in this gene may indirectly propagate the development of alcohol dependence by impairing homocysteine metabolism via inhibiting methionine synthase.

Findings of the present study and available literatures suggest that* MTHFR* C677T polymorphism influence the etiology of alcohol dependence. However, focusing on the ill effects of* MTHFR* C677T gene polymorphism and its interaction with alcohol would be more meaningful rather than focusing on the development of alcohol dependence process. However, present study has some major limitations like smaller sample size (low study power) and lack of data on related biochemical variables including homocysteine levels, folate, and vitamin B_12_. Further studies are required to understand the exact pathophysiological mechanism of the development of alcohol dependence with respect to* MTHFR* C677T gene polymorphism. Therefore, association of* MTHFR* 677TT genotype with hyperhomocysteinemia, folate, and vitamin B_12_ levels could assist physicians in identifying AD patients as well as improvement in addiction management.

## 5. Conclusions

In brief, to our knowledge, this is the first study attempted to understand the extent of* MTHFR* C677T gene polymorphism against alcohol dependence in Indian context. The present study indicates that* MTHFR* C677T polymorphism has no direct significant conferring risk for AD among* Meiteis* of Manipur. However, the selected polymorphism may directly or indirectly affect the one-carbon metabolism by interacting with environmental risk factors and subsequently progressing towards the development of AD. Higher prevalence of the mutant allele in the studied population may exacerbate deleterious future health risk among alcohol drinkers (especially AD cases). Therefore, the present paper paves a way for further population specific studies specifically interaction of selected SNP with biochemical variables including homocysteine, folate, vitamin B_12_, liver function test, etc. to understand its relationship and predictive outcomes of health effects as well developing right interventional strategies and therapeutics.

## Figures and Tables

**Table 1:** Characteristic feature of the selected SNP *MTHFR* C677T polymorphism.

Gene	SNP (rs)	Position	Nucleotide sequence	Restriction enzyme	Reference
*MTHFR *	C677T (1801133)	Exon 4	F5′TGAAGGAGAAGGTGTCTGCGGGA3′	*Hinf*I	Arruda et al., 1997 [16]
			R5′AGGACGGTGCGGTGAGAGTG3′		

**Table 2:** Genotypic and allelic frequency distribution of *MTHFR* C677T gene polymorphism among different patterns of alcohol consumption.

Genetic Marker (*MTHFR* C677T)	AD cases (*N* = 139)	Controls (ND + OD + MD) (*N* = 313)	ND (*N* = 121)	OD (*N* = 113)	MD (*N* = 79)	*χ* ^2^ (*P* value)			
						*P* ^1^	*P* ^2^	*P* ^3^	*P* ^4^
CC (%)	91 (65.47)	228 (72.84)	89 (73.55)	81 (71.68)	58 (73.42)	0.283	0.366	0.876	0.352
CT (%)	44 (31.65)	78 (24.92)	29 (23.97)	28 (24.78)	21 (26.58)				
TT (%)	4 (2.88)	7 (2.24)	3 (2.48)	4 (3.54)	0				

C (frequency)	226 (0.81)	534 (0.85)	207 (0.86)	190 (0.84)	137 (0.87)	0.129	0.334	0.901	0.378
T (frequency)	52 (0.19)	92 (0.15)	38 (0.14)	36 (0.16)	21 (0.13)				

*N*: number; ND: nondrinkers; OD: occasional drinkers; MD: moderate drinkers.

*χ* ^2^: likelihood ratio test comparing genotype and allele frequency; *P* ^1^ = AD versus control (ND + OD + MD); *P* ^2^ = AD versus ND; *P* ^3^ = ND versus OD; *P* ^4^ = ND versus MD; significance level at 5%.

**Table 3:** Relative risk analysis (Odds Ratio) of *MTHFR* C677T polymorphism and different drinking patterns.

*MTHFR* C677T	AD	Control	ND	OD	MD	OR^1^ (95% CI)	*P* ^1^	OR^2^ (95% CI)	*P* ^2^	OR^3^ (95% CI)	*P* ^3^	OR^4^ (95% CI)	*P* ^4^
CC	91	228	89	81	58	Reference		Reference		Reference		Reference	
CT	44	78	29	28	21	1.41 (0.91–2.20)	0.12	1.48 (0.85–2.58)	0.16	1.06 (0.58–1.93)	0.84	1.11 (0.58–2.13)	0.75
TT	4	7	3	4	0	1.43 (0.41–5.01)	—	1.30 (0.28–5.99)	—	1.46 (0.32–6.74)	—	—	—
CT + TT	48	85	32	32	21	1.41 (0.92–2.17)	0.11	1.47 (0.86–2.50)	0.16	1.10 (0.62–1.95)	0.75	1.01 (0.53–1.91)	1

ND: nondrinkers; OD: occasional drinkers; MD: moderate drinkers.

OR^1^: odd ration between AD and control; OR^2^: odd ration between AD and ND; OR^3^: odd ration between ND and OD; OR^4^: odd ration between ND and MD; CI: confidential interval.

*P* ^1^ = AD versus control (ND + OD + MD); *P* ^2^ = AD versus ND; *P* ^3^ = ND versus OD; *P* ^4^ = ND versus MD; significance level at 5%.
